# Cutting-edge advances in modeling the blood–brain barrier and tools for its reversible permeabilization for enhanced drug delivery into the brain

**DOI:** 10.1186/s13578-023-01079-3

**Published:** 2023-07-27

**Authors:** Amit Sharma, Diogo C. Fernandes, Rui L. Reis, Dominika Gołubczyk, Silke Neumann, Barbara Lukomska, Miroslaw Janowski, Marcin Kortylewski, Piotr Walczak, J. Miguel Oliveira, Jarek Maciaczyk

**Affiliations:** 1 Department of Stereotacitc and Functional Neurosurgery, University Hospital Bonn, 53127 Bonn, Germany; 2 3B’s Research Group, I3Bs—Research Institute on Biomaterials, Biodegradables and Biomimetics, University of Minho, Headquarters of the European Institute of Excellence on Tissue Engineering and Regenerative Medicine, AvePark, Parque de Ciência e Tecnologia, Zona Industrial da Gandra, 4805-017 Barco, Guimarães, Portugal; 3 ICVS/3B’s—PT Government Associate Laboratory, 4710-057 Braga, Portugal; 4 Ti-Com, Polish Limited Liability Company, 10-683 Olsztyn, Poland; 5 Center for Translational Medicine, Warsaw University of Life Sciences, 02-797 Warsaw, Poland; 6 Department of Pathology, University of Otago, Dunedin, 9054 New Zealand; 7 NeuroRepair Department, Mossakowski Medical Research Institute, Polish Academy of Sciences, 02-106 Warsaw, Poland; 8 Department of Diagnostic Radiology and Nuclear Medicine, University of Maryland School of Medicine, Baltimore, MD USA; 9 Department of Immuno-Oncology, Beckman Research Institute at City of Hope Comprehensive Cancer Center, Duarte, CA 91010 USA; 10 Department of Surgical Sciences, University of Otago, Dunedin, 9054 New Zealand

**Keywords:** Blood–brain barrier, Drug targets, In vitro models, In vivo models, Drug delivery, Organoid models, Focused ultrasound, Intra-arterial infusion

## Abstract

The blood–brain barrier (BBB) is a sophisticated structure whose full functionality is required for maintaining the executive functions of the central nervous system (CNS). Tight control of transport across the barrier means that most drugs, particularly large size, which includes powerful biologicals, cannot reach their targets in the brain. Notwithstanding the remarkable advances in characterizing the cellular nature of the BBB and consequences of BBB dysfunction in pathology (brain metastasis, neurological diseases), it remains challenging to deliver drugs to the CNS. Herein, we outline the basic architecture and key molecular constituents of the BBB. In addition, we review the current status of approaches that are being explored to temporarily open the BBB in order to allow accumulation of therapeutics in the CNS. Undoubtedly, the major concern in field is whether it is possible to open the BBB in a meaningful way without causing negative consequences. In this context, we have also listed few other important key considerations that can improve our understanding about the dynamics of the BBB.

## Basic architecture and key molecular constituents of the blood–brain barrier

The complex organization of the blood–brain barrier (BBB) is not only attributed for the exchange of passive diffusion/efflux of solutes in the blood or for the active transport of nutrients to the brain, but also for regulating the migration of circulating immune cells. Of interest, the dynamic association of microvascular endothelial cells (ECs) with pericytes, astrocytes and microglia, together with their specialized structural composition of tight junctions (TJs)/adherens junction (Ajs) form the main interface for intracellular signaling. A wealth of literature published during the last decades has evidenced a strong correlation between BBB dysfunction, alteration of TJ complexes and progression of multiple CNS diseases (e.g., stroke, multiple sclerosis, brain tumors, neuroinflammatory and neurodegenerative diseases). Given that BBB alterations have been identified in major depressive disorder, bipolar disorder and schizophrenia, a recent study discussed that gender differences exist in inflammation-induced loss of BBB integrity and that BBB-related transcriptional changes occur differently in men and women [1]. Under certain conditions, the BBB appears to adapt to the needs of the CNS, specifically relating to the passage of relevant proteins. For instance, an interesting study showed that radiolabeled alpha-synuclein (a small protein in Lewy bodies, linked to Parkinson’s disease) traverses the BBB bidirectionally, i.e., toward both brain-blood and blood–brain at rates consistent with saturable mechanisms [2]. Besides alpha-synuclein, amyloid beta-peptides and prion proteins have also been discussed for crossing the BBB, whereas the possibilities of tau proteins to bidirectionally cross the BBB have been discussed [3, 4].

Similarly, it has been shown that a possible transport of activated protein C across the mouse blood–brain barrier requires an efficient Endothelial protein C receptor [5].There have been continuous efforts to establish the causal relationship between disease-related mutations and BBB impairment. For instance, a recent study demonstrated that mutations associated with neurodegenerative diseases can independently cause BBB dysfunction [6].It is an undeniable fact that the genomic mutation data from several models have enhanced the spectrum of BBB. For instance, the loss-of-function mutations in the NIMA-Related Kinase 1 (*NEK1*) gene, which encodes a serine/threonine kinase, are involved in human developmental disorders and amyotrophic lateral sclerosis (ALS). A recent study showed that the metabolic dysfunction in Nek1 deficient cells reduces the levels of A20 (an important ubiquitin editing enzyme) to promote the activation of RIPK1 (Receptor Interacting Serine/Threonine Kinase 1), necroptosis of CD31+ endothelial cells and BBB damage [7]. There have also been indirect evidences, such as P-glycoprotein (Pgp), encoded in the ATP-binding cassette B1 (*ABCB1*) gene expressed highly at BBB, and a study has shown that single nucleotide polymorphisms (SNPs) in *ABCB1* may contribute to the progression of amyloid beta deposition in the brain [8]. In context to epigenetic mediators of BBB, there have been limited data. Among them, Kalani et al. put forward an interesting hypothesis about the miR29b-induced mechanism of BBB dysfunction. The authors proposed that miR29b directly targets DNMT3b (DNA Methyltransferase 3 beta), which in turn regulates MMP9 (Matrix metallopeptidase 9) levels. Because MMP alters junctional proteins (e.g., occludens, claudins, and cadherins), this leads to an impact on BBB permeability [9]. In fact, miRNAs (miR-150, miR-212, miR-132, miR-501-3p, miR-96, miR-424-5p, miR-101, miR-181a) have been found to modulate physiological and pathological processes by regulating TJs and ultimately affecting the integrity/permeability of the BBB [10]. Besides DNMTs, histone deacetylases (HDACs), which catalyze the deacetylation of histone proteins and thus inhibit transcription and gene expression, have also been linked to the BBB. For example, histone deacetylase-6 inhibitors (HDAC6is) that penetrate the blood–brain barrier have been discussed as a potential strategy for the therapy of CNS disorders [11]. Here, it is also important to mention the role of caveolin-1, which can protect the integrity of the BBB by inhibiting matrix metalloproteinases (MMPs) that degrade TJ [12, 13]. Recently, a study described that over-expression of Mfsd2a (major facilitator superfamily domain containing 2a) attenuates BBB dysfunction via the caveolin-1/Nrf-2/HO-1 pathway [14].

Recently, a study unraveled a number of key players involved in the interaction between breast cancer cells (BCCs) and BBB endothelial cells that underlie BBB alterations and transendothelial migration of malignant cells [15]. Over the past decade, studies have shown several signaling pathways required not only for BBB formation but also for BBB integrity and function, among them Wnt/β-catenin, retinoic acid and sonic hedgehog pathways emerged as the focus of BBB research [16, 17]. A recent study demonstrated that the endothelial transmembrane receptor Unc5B and its ligand netrin-1 regulate BBB integrity by maintaining Wnt/β-catenin signaling [18]. Despite decades of research, the complete picture of the dynamic mechanism or regulators that play a competitive (protective or disruptive) role for BBB integrity remains elusive.

## In vitro BBB modeling and drug studies

The concept of in vitro models is born with the surge of tissue engineering, despite the previous use of cells onto plastic surfaces for early cytotoxicity testing of compounds of interest. Modelling is the ability to mirror the characteristics of a complex system, with the purpose of extracting information from it. In health sciences there are four main types of models: in vivo (live animals)*, *ex vivo (lab cultured resected tissue)*, *in silico (computer models) and, in vitro*. *In vitro models are a bioengineering effort to mimic the tissue of interest based on the triad stem cells, extracellular matrix, and soluble factors. Applied to the development of in vitro neurovascularity unit, this concept has resulted in several models that can recapitulate main features of BBB, with the most recent advances and developments being reviewed in the following sub-section.

### BBB in vitro models

In recent years, the interest in the development of in vitro models to recapitulate the human BBB has increased. The search for models that can reduce the number of animals used for research has led to the development of new techniques and contributed to the growth of the biotechnology industry, particularly the industry of microphysiological models (MPS). The main application of MPS is in the development of new drugs or new drug delivery strategies. Following this trend, several high-profile investigators have pushed the use of these models and proposed a roadmap for the integration of MPS in the drug development industry [19]. The creation of BBB models is imperative when considering targeting drug delivery to the CNS and has led to the development of several models over the recent years. Fernandes et al. have recently reviewed these advances and some of the controversial standards used in the field [20]. In neurovascular models, and almost every other model, there are undoubtedly game changers that have led to the implementation of standards for new models. The drug delivery field has been developing for 3 decades but has fallen short of its potential. This shortcoming can be explained by the duality faced by this field: when the tissue is available, the conservative approach works and the motivation for innovative systems is null; if the tissue is unavailable, the task of directional and localized delivery is complex. The development of an effective drug delivery system has been halted by the absence of testing platforms that presented an in vivo*-*like challenge [21]. The use of in vitro BBB models has become widespread and new drug delivery systems targeting the brain are currently tested in in vitro models. However, the validity of these models is often questioned, raising doubts about the translation of the results. Some of those doubts have been dissipated, since a monolayer of brain endothelial cells has been shown to have a similar permeability for positron-emitting tomography (PET) radioligands as the BBB in human patients [22]. In vitro BBB models consist of differentiated or primary cells assembled in a predefined ratio to create a functional liquid-tissue barrier. The presence of astrocytes and pericytes is crucial for obtaining an impermeable in vitro BBB model [23].

### Organoid-based BBB models

Organoids are self-organized cellular structures that can be derived directly from patient tissue or through the use of developmental biology. Organoids exhibit characteristics of several organs such as the pancreas, gut, retina, and brain [24]. Researchers have aimed to standardize procedures while pushing for an increased diversity of tissues within each organ, particularly the brain. Models of different brain areas exist, namely the cortex [25], choroid plexus [26], and thalamus [27]. In the latter model [27], organoids from the thalamus and cortex are fused, mimicking the in vivo interplay between these two brain areas. CNS-based organoids show native tissue-like features, such as complex electrical activity [28], selective transport of molecules across the liquid-tissue barrier [26] and production of cerebrospinal fluid (CSF). However, despite these interesting advances, the absence of vascularization in these brain organoids limits their potential as drug testing platforms. In situ drug administration in the CNS, particularly in the brain, is a challenging procedure, mostly destined to require surgery [29, 30]. Systemic administration is the most widely used drug administration technique, despite intranasal [31] and intrathecal [32] administration having shown promise for spinal cord related treatments.

The presence of a BBB in organoids is crucial for the design of a fully biomimetic drug or disease testing platform. The need for these features has been recognised by the leaders in the field and was reviewed extensively in 2018 [30]. Vascular structures that allow perfusion are essential to represent a blood-tissue interface. Thus, the logical step using organoids was to develop BBB organoids that could be integrated into brain organoids. Simmoneau et al. developed a new high-throughput method to produce homogeneous and precisely characterized BBB organoids [33]. The scale-up was achieved using a Gri3D, a micropatterned hydrogel well plate that allows rapid and consistent organoid formation and growth with low heterogeneity. Meant to keep the organoid in suspension within microcavities, this system relies on the absence of an adhesive matrix to achieve highly homogeneous organoids [34]. High precision image acquisition and processing are implemented to characterize the BBB organoids. Functional BBB organoids have to exhibit three cell layers—endothelial cells, pericytes and astrocytes to ensure highly selective permeability of substances across this membrane. Showing impermeability to dextran particles ranging from 4 to 70 kDa and with values of transendothelial electrical resistance (TEER) over 2000 cm^2^, these organoids are comparable to other BBB models [20]. The 3 layers can be visualized using immunocytochemistry and show astrocytes at the core, a pericyte shell and an outward layer of brain endothelial cells. The organoid diameter is approximately 200 mm for 24-wells and 96-wells Gri3D, showing consistency in the formed organoids. The functionality of the BBB organoids was assessed by measuring transferrin-specific transcytosis. The authors use anti-transferrin receptor antibodies to cross the BBB-organoids, showing that transferrin-mediated transport is clathrin- dependent, which can help develop new drugs to target this crossing [33].

**Fig. 1:**
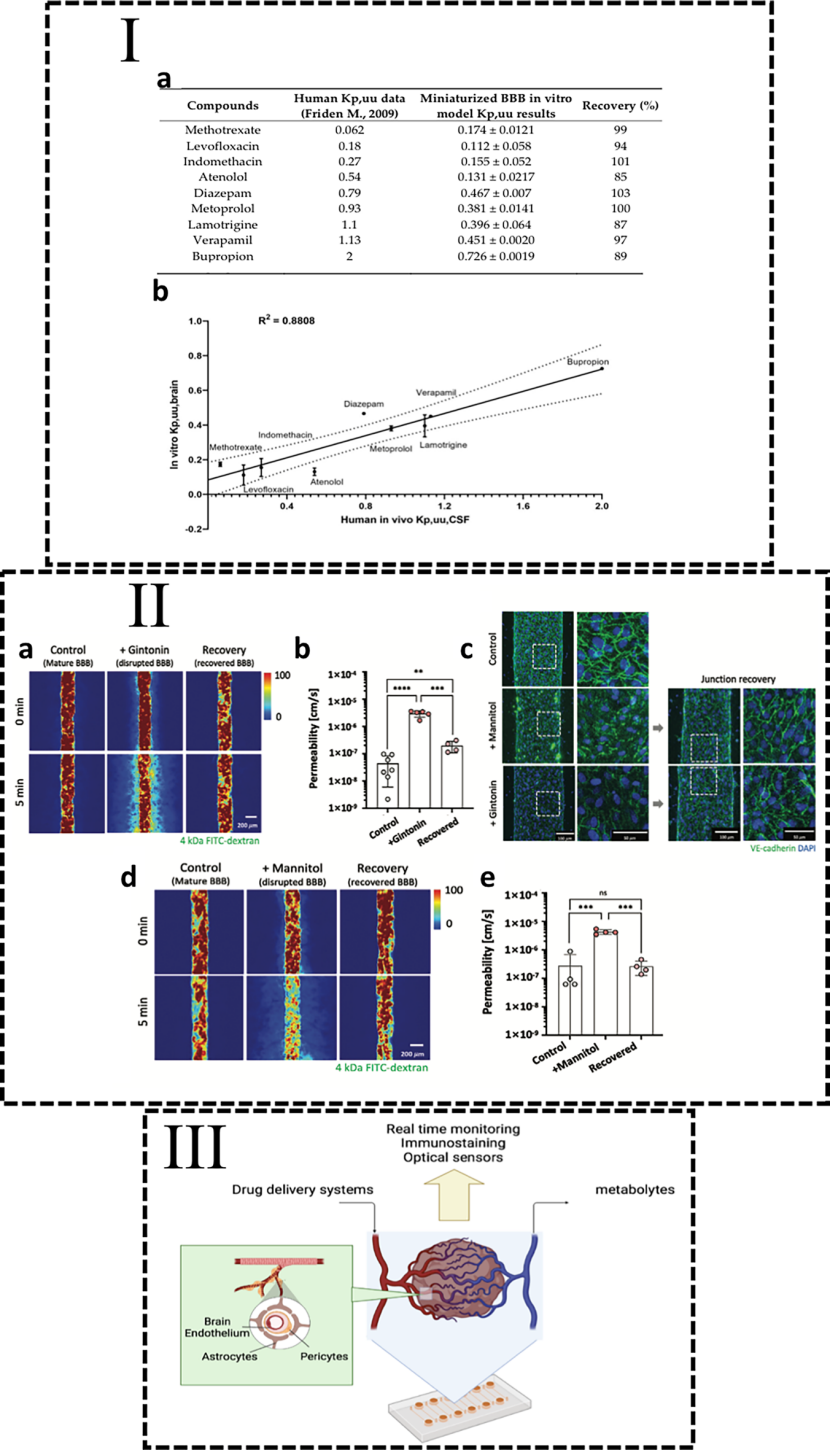
In vitro BBB models’ permeability translation and drug testing future perspective. **I**-**a** Permeability measurements measurement techniques can allow a profiling of the permeability of a library of drugs which can be compared to in vivo values, and **b** correlated, showing the validity of these models. **II** Possible future drug testing hybrid platforms, using assembled vascular networks and brain organoids. **III** is adapted from [55], respectively

BBB organoids can be fused with cerebral organoids to form neurovascular structures. These structures exhibit BBB features such as basement membrane specific proteins and vessel-like morphology whilst cerebral organoids express standard neuronal markers [35]. Neurovascular organoids can be prepared without a previous separate and independent differentiation [36, 37]. However, the limitation of these systems as compared to the simpler BBB organoids is the absence of a blood-tissue barrier, since the vasculature still does not allow perfusion. The employment of microfluidic or rapid manufacturing in combination with knowledge from the field of biomaterials can provide solutions to the problem of absence of a functional vasculature. By means of using microfluidics, cerebral organoids can be integrated in a microvasculature of human umbilical vein endothelial cells (HUVECs), allowing vasculature-based perfusion through the organoid [38]. The robust and reproducible integration of organoids into functional vascular structures is a major step towards a platform that allows personalized drug testing for neurodegenerative diseases (Fig. 1).

### Organoid-absent BBB models

Innovations in the biomedical field that do not follow the state-of-art biological standards, usually showcase a technological progress or methodology that makes them more translatable, due to the robustness of the process, a monitoring capacity or the relevance of the application of the model itself. Since the organoids’ field is still young and requires maturation, applied works often follow simpler biological approaches to hint at new models, from which straightforward outcomes can be extracted. Examples of this paradigm are models that test glioblastoma’s drug sensitivity and therapy strategies [39] or Sars-Cov-2 infection effects over brain’s permeability and physiology [40]. These models contribute with hints at the consequences and possible therapies for the health problem working as magnifying lenses on the tissue of interest. Whilst the modeling the brain’s infection by Sars-Cov-2 virus hints that BBB disruption can be one cause for the neurologic symptoms felt by several COVID-19 patients [40], in the assembling of the glioblastoma neurovascular model, it is possible to observe an increased aggressiveness of the model implanted tumor in the presence of the BBB. This way, it shows the significance of having neurovascular models even for the in vivo-like behavior of an in vitro grown tumor. However, more importantly, this model proposes a new method for improved glioblastoma treatment using conventional chemotherapy, by using transferrin-modified porous silicon nanoparticles as BBB transposing drug carriers. Using doxorubicin, it allows the use of conventional chemotherapy for effective glioblastoma multiforme treatment, alternatively, the use of a therapeutic drug that can simultaneous cross BBB and treat the tumor [39]. The potential use of conventional chemotherapy for the treatment of brain tumors can revolutionize a field that has felt a halt for nearly 20 years.

However, most drug tests performed using BBB platforms focus on advanced drug delivery strategies, such as summarized in Table 1. These are often centered on transcytosis, or shuttling, through the BBB, ignoring biophysical factors that can lead to more effective drug delivery. Interestingly, the shape of the nanoparticles significantly influences the crossing of the endothelial barrier. Despite being in a brain endothelial monolayer, rod-shaped particles permeate through it 1.5 times more than isotropically shaped particles, showing another angle to enhance drug delivery through highly impermeable endothelial barriers [41]. Even though testing of external agents, such as tumors, viruses or bacteria are of profound interest, BBB disruptions are by itself a pathology with severe consequences on the neurologic state of individuals [42]. Therefore, modeling of BBB pathologies and disruptions is a priority in the field. Brain ischemia is one of the two main concerns regarding BBB disruptions, particularly considering brain-related vascular problems are a main health concern worldwide [43]. Modeling of brain ischemia in vitro shows increased permeability of endothelial cells due to oxygen deprivation with astrocytes and pericytes potentiating the leakage of vessels upon ischemia-derived oxygen deprivation. This increased leakage can cause more systemic neurologic consequences than the localized oxygen deprivation [43]. Disruptions of the BBB have also been closely linked to aging-related diseases such as neurodegenerative diseases. Despite the ignorance about whether it is a cause or a consequence, disruption of the BBB is a hallmark of degeneration [44]. Alzheimer’s disease, one of the most concerning forms of dementia, can currently be modeled using a microfluidic chip, recapitulating the neurovascular hallmarks of this disease and allowing a complete molecular characterization of the key players in the degeneration process [45]. Building on this model, if a complete BBB instead of a simple endothelial barrier is used, this platform can potentially become a highly relevant tool in the search for innovative Alzheimer’s disease therapies, and drug development and testing. Simpler models have been used for such purposes, but translation to a diseased tissue seems an uncertain extrapolation without the presence of a complete and fully characterized BBB [46]. The absence of organoids does not imply a disregard for developmental biology, nor that the intrinsic biology of BBB or neurovascularity is secondary to the technological advances. Using a hypoxia-based differentiation step, human induced pluripotent stem cells (hIPSC)-derived brain endothelial cells assembled together with primary astrocytes and pericytes in a microfluidic chip have comparable permeability of BBB-crossing drugs to the ones measured in vivo [47]. There is an urgent need for neurovascular models, recapitulating the main features of the BBB, that can bypass the problem of the drug delivery to the CNS and the brain. The combination of advanced assembling strategies of organoid-absent models with the biological accuracy of neurovascular organoids can create complex yet effective drug testing platforms. Considering the resemblance with human native tissue and the level of complexity, these models have the potential to revitalize the drug discovery process unlike what the use of animal models was able to accomplish in recent decades.

**Table 1:** Drug delivery systems tested in BBB in vitro models

Present BBB layers	Cell types and origin	Drug delivery system	Therapeutic molecule	Results	Refs.
1	hCMEC/D3 brain endothelial cell line	Lipid nanocapsules	Nonpsychotropic cannabinoids	This system outperformed G-Technology by sixfold crossing the in vitro BBB, a sustained drug delivery system to the brain currently in the last stage of clinical trials	[48]
	hIPSCs-derived brain endothelial cells	None	Immunoglobulin G	The antibody transport across the in vitro barrier is increased in the presence of amyloid-b and neuroinflammatory cytokines, proving to be a plausible therapy for Alzheimer’s disease	[49]
	Primary human microvascular endothelial cells	None	Anti-amyloid-b monoclonal antibody	Anti-amyloid-b N-terminal antibodies allow clearance of amyloid-b across the BBB unlike Anti-amyloid-b C-terminal, due to a RAGE-based process	[50]
	Bovine primary brain endothelial cells	Phage display-based HAIYPRH sequence containing peptides	None	MALDI-TOF allowed a more sensitive analysis of the BBB crossing peptides, allowing highly sensitive and low sample assays for peptide selection	[51]
	hIPSC’s derived brain endothelial cells	None	Cyclosporine A	Evaluation of Cyclosporine A time and concentration dependent toxicity using a simple in vitro BBB model	[52]
	hIPSC’s derived brain endothelial cells	None	Mannitol	The miniaturized in vitro model of BBB allows the visualization and characterization of the hyperosmotic permeability enhancement caused by mannitol dosages	[53]
	hCMEC/D3 brain endothelial cell line	Recombinant human apoferritin nanoparticles	Anti-CTX and anti- TZ monoclonal antibodies	Using a glioblastoma cell line and a well plate insert BBB model, ferritin-based nanocarriers of monoclonal antibodies show BBB crossing capacity and posterior glioblastoma targeting	[54]
	hCMEC/D3 brain endothelial cell line	Spheric and rod-shaped polystyrene particles	None	Rod-shaped particles overcome the BBB more easily than spherical particles of the same material	[41]
2	Primary rat endothelial cells and astrocytes	Phage display derived peptides	None	The two selected peptides show particularly high BBB permeability as compared to control ones with 3.3 × 10^−7^ cm/s for GLHTSATNLYLH and 1.5 × 10^−6^ cm/s for VAARTGEIYVPW	[41]
		GLHTSATNLYLH and VAARTGEIYVPW for primary rat endothelial cells			
	Human CD34 + endothelial cells derived from hematopoietic stem cells	None	Methotrexate Levofloxacin Indomethacin Atenolol Diazepam Metoprolol Lamotrigine Verapamil Bupropion	The blood/cerebrospinal fluid (CSF) ratio for each of the used drugs matched the values acquired for this ratio in human patients with a correlation factor R^2^ = 0.88	[55]
	Murine immortalized endothelial cells and murine astrocytes	Transferrin and cell penetrating peptide PFVYLI modified liposomes	Doxorubicin and erlotinib	Dually modified liposomes showed significantly higher BBB crossing ability and glioblastoma cells’ death than controls	[56]
3	hIPSCs derived brain microvascular endothelial cells and primary brain pericytes and astrocytes	100–400 nm fluorescent polystyrene and 100 nm rhodamine-labeled polyurethane nanoparticles	None	The BBB vascularized microfluidic model allows rapid nanoparticle testing, concluding that both the commercial polystyrene and the custom-made polyurethane nanoparticles have similar BBB permeability	[57]
	hIPSCs derived brain microvascular endothelial cells and primary brain pericytes and astrocytes	None	Digoxin Colchicine Quinidine Vinblastine Glibenclaide Caffeinem Bupropion Gabapentin Lamotrigine Tacrine Thioridazine Topiramate Verapamil	Higher correlation of in vitro human BBB model crossing with human in vivo models of standard drugs than rat BBB and Caco-2 models	[58]
	Immortalized human brain endothelial, brain pericytes and astrocytes	None	Propranolol Pyrilamine Memantine Diphenhydramine Sodium fluorescein Lucifer yellow	Immortalized BBB recreated the in vivo permeation profile of BBB permeable and non-permeable drugs	[59]

## Animal studies on BBB permeability

### In vivo* models and optimal techniques*

In vitro BBB modeling has clear advantages such as cost effectiveness and high throughput, but due to the complexity of the BBB, the available models are far from perfect and thus animal models are still a mainstay of research into the physiology, pathology and controlled manipulation of the BBB. In vivo models provide unique insight into the cellular, morphological and functional properties and barrier permeability in healthy and disease-damaged brains. Of note is that some evidence suggests functional differences between human and rodent BBB with the latter characterized by lower threshold for disruption [60]. Numerous strategies aiming at controlled and transient permeabilization of the BBB are currently being developed to intensify drug or therapeutic cell transport across the BBB for their effective accumulation in the brain. Several techniques have been developed for opening the BBB, ranging from the use of chemical and biological substances, osmotic opening, to physical stimuli such as focused ultrasound with systemically administered microbubbles. Below we present characterization and preclinical applications of the most commonly used techniques (Table 2).

**Table 2:** Animal models of BBB

Method	Species	Number of animals	Molecule type	BBBO Readout
Osmotic	Rat [61]	Not specified	Evans blue	Evans blue
	Rabbit [62]	n = 65	Evans blue	Evans blue
	Monkey [63]	n = 28	Evans blue	Evans blue
	Rabbit [64]	Not specified	Evans blue	Evans blue
	Dog [65]	n = 38	Methotrexate	Evans blue
	Rat [66]	n = 64	Herpes simplex virus, (HSV), and paramagnetic monocrystalline iron, oxide nanoparticles (MION)	MRI
	Rat [67]	n = 152	Methotrexate	Evans blue albumin and quantitatively by measuring, delivery of the low molecular weight marker [3H]-methotrexate
	Rabbit [68]	n = 23	Evan’s Blue	Evan’s Blue
	Rabbit [69]	n = 8	Evan’s Blue	MRI
	Mouse [70]	n = 38	Monoclonal antibody	MRI, microscopy
	Mouse [71]	n = 12	Monoclonal antibody (^89^Zr-BVDFO)	PET/CT Imaging
	Mouse [72]	n = 32	Rhesus, macaque derived adeno-associated viral (AAV) vector	MRI
MAP	Mice [73]	Not specified	Beta-galactosidase	Histology
	Mouse [74]	Not specified	Cisplatin, methotrexate	Evans blue/Crocein Scarlet/Light Green SF
	Mouse [75]	n = 43	Melittin	MRI and Evans blue staining
VEGF	Rat [76]	n = 25		FITC-dextran
	Mouse [77]	n = 27	Evans blue	MRI, Evans blue staining
VEGF intraspinal injection	Rat [78]	n = 9	Evans blue	Evans blue staining
	Rat [79]	n = 25	MOG1-125 peptide	MRI, IHC
FUS	Rabbit [80]	n = 22	Albumin coated microbubbles	MRI
	Rat [81]	n = 47	5 Different magnetic resonance contrast agents	MRI
	Mouse [82]	n = 15	A rabbit anti-human, dopamine D4 receptor antibody	MRI, Trypan Blue
	Rat [83]	n = 83	Doxorubicin	MRI
	Mouse [84]	n = 44	MGPP3 cells	MRI
	Mouse [85]	n = 28	Monoclonal antibody (mCD47)	PET/CT imaging
	Mouse [86]	n = 52	Anti-pGlu3 Aβ mAb	fluorescent images of Trypan blue delivery
	Rat and Mouse [87]	n = 42 and n = 16, respectively	Doxorubicin/Evans Blue	MRI, Evans Blue injection
	Rat [88]	n = 4–6 per group	Polymeric nanoparticles	Evans blue, MRI

#### Osmotic BBB opening (OBBBO)

Rapoport first described in the early 1970s that infusion of hypertonic substances such as arabinose, urea or mannitol causes endothelial cells to contract, thereby increasing vascular permeability, effectively resulting in transient opening of the BBB [89]. Since then, the technique has been widely utilized in animals and in patients suffering from brain cancer. The method in small animals (mice, rats) is relatively invasive as it requires gaining surgical access to the internal carotid artery. Procedure starts with skin incision in the area of muscle triangle on the neck of the animal to expose carotid arteries. Extracranial branches (external carotid artery, occipital artery and the pterygopalatine artery) are ligated to route the entire flow into cerebral arteries. Then a small arteriotomy is made for catheter placement either into the common carotid artery (CCA) with permanently disrupted perfusion of the ipsilateral CCA, or into the external carotid artery and with preserved perfusion of the CCA. With vascular access to cerebral arteries a short bolus (40–60 s) of hyperosmotic mannitol (25%) is infused, displacing the blood and leading to BBB opening in brain regions supplied by the catheter infusion. Early work by Rapoport et al. showed that OBBBO can be used with success in various species including rats [61] rabbits [62] and primates [63].

BBB breach was assessed by intravenous injection of Evans blue in Ringer solution immediately after OBBBO and demonstrated cerebral accumulation of the blue dye. In 1973 Brightman et al. used electron microscopy to show that 3 M urea led to opening of endothelial tight junctions [90]. Burks et al. demonstrated that BBB disruption with mannitol can be exploited for immunomodulation as it results in increased production of cell-signaling proteins [91]. However, the main motivation for BBB opening has been to improve accumulation of drugs in the brain that would otherwise have no access to their targets behind the BBB. The OBBBO method was applied to enhance penetration of systemically injected methotrexate in dogs and indeed, drug accumulation improved tenfold [65]. Significant advancements with mannitol-based osmotic techniques have been made by the group of Dr. Neuwelt, developing protocols for intra-arterial administration of chemotherapeutics, viral vectors or nanoparticles [66]. Dr. Neuwelt’s group reported in 1999 that reliability of the OBBBO is affected by multiple factors such as the partial pressure of CO2 (PaCO2) in the blood, the choice of anesthetic and other factors [67]. Indeed, one of the major disadvantages of the OBBBO and the reason why it has not found broad clinical adaptation is the high variability of the area of BBB disruption [68]. This high variability was the main motivation for developing imaging technology that would allow performing BBB opening with high precision and reproducibility. Progress in magnetic resonance imaging (MRI) technology with MRI-compatible interventional instrumentation and particularly improved temporal resolution has enabled MRI-guided neurointerventions that have proved particularly useful for improving reliability of osmotic BBB opening (OBBBO). Other imaging modalities such as intravital two photon microscopy (2 PM) or positron emission tomography (PET) imaging have also helped to guide intra-arterial drug delivery. Foley et al. used dynamic contrast enhanced (DCE)-MRI to verify the territory of OBBBO after mannitol infusion followed by intra-arterial injection of adeno-associated virus (AAV) vectors. They showed for the first time that a single administration of AAV vectors provides widespread transgene production in brain tissue [72]. Progress in interventional neuroradiology has resulted in renewed interest in intra-arterial drug delivery and more advanced imaging protocols are being developed to predict territory of OBBBO as shown by Janowski et al. in a rabbit model [69]. Chu et al., introduced a reproducible method of BBB opening in mice under the guidance of both, MRI and multi-photon microscopy. With this dynamic multi-modality imaging, intra-arterially administered antibodies were shown to cross the osmotically opened BBB and accumulate in the brain [70]. Moreover, Lesniak et al. showed with dynamic PET imaging that OBBBO strongly enhanced uptake of an intra-arterially administered imaging agent (89Zr-BVDFO) in naïve mice while intravenous administration resulted in negligible brain accumulation of the imaging agent regardless of the BBB status [71]. The same phenomenon was also observed for nanobodies, while dendrimers failed to benefit from intra-arterial delivery following OBBBO [92].

#### Membrane active peptides

Membrane active molecules are a group of substances that interact with cell membranes leading to their destabilization and increased permeability [93]. Sarkar et al. have recently reported a carrier peptide (K16ApoE) that facilitates transport of various proteins and immunoglobulins across the BBB in a non-covalent manner [73]. This study was followed by a report showing that K16ApoE led to transient BBB disruption and enabling passive transport of other (non-ligand) molecules [74]. Another membrane active peptide naturally occurring in honeybee venom is melittin. It has been shown that melittin triggers reversible destabilization of cell–cell junctions and disruption of barrier function in in vitro BBB model. In mice, intra-arterial injection of 3 μM melittin resulted in robust and reversible BBB opening. Of note, injection of 5 μM peptide led to neurological deficits indicating a narrow therapeutic window [75]. Recently cyclic guanosine monophosphate–quality version of the natural monoterpene perillyl alcohol (NEO100) studied as anti-glioma agent has been shown to effectively disrupt blood brain barrier [94]. Intra-arterially injected NEO100 intercalates into cell membranes of endothelial cells causing their transient destabilization and resulting BBBO lasting several hours.

#### Vascular endothelial growth factor

Vascular endothelial growth factor (VEGF), also known as vascular permeability factor, is a signaling polypeptide produced by many cells that regulates function of blood vessels and is best known for stimulating the formation of blood vessels [95]. VEGF, when applied topically to the cerebral microcirculation, triggers an increase in the permeability of the BBB to FITC-dextran-10K and dilates cerebral arterioles [76]. Changes in BBB permeability were also observed after intravenous injection of VEGF [77]. VEGF-based opening of the BBB through stereotaxic injection into the spinal cord parenchyma has been used as a method to induce focal demyelination in rats immunized against myelin antigens [78]. A similar strategy has been used to model multiple sclerosis in the rat brain [79].

#### Focus ultrasound

One of the recent advances in BBB opening is the mechanical destabilization of tight junctions within the cerebral endothelium. The technique uses low-frequency ultrasound waves in combination with intravenously injected microbubbles. Microbubbles in cerebral vasculature amplify local cavitation resulting in BBB breach. This method is spatially selective, relatively straight forward and non-invasive. The first reports of the use of this method date back to the beginning of the twenty-first century. In a study by Hynynen et al. in 2005, the feasibility of transmitting focused ultrasound (FUS) energy across the intact rabbit skull was assessed [80]. The group showed that FUS with frequency of 0.69 MHz resulted in BBB disruption in the sonicated brain area. However, observation with an electron microscope showed a few cases of subtle endothelial damage. A study by Marty et al. focused on dynamic imaging of BBB closure after FUS and assessed the size of the pores resulting from standard FUS procedure. Molecules with an average size of about 1 nm were able to pass freely through the barrier for more than 10 h, whereas larger iron oxide nanoparticles (> 25 nm) were able to do so for only a few minutes after sonication [81]. One of the prime applications for FUS BBB opening is to improve brain accumulation of therapeutics for treatment of neurological brain tumors. Doxorubicin, an anti-cancer agent, was shown to accumulate in the sonicated hemisphere and remained significantly higher than in the contralateral non-treated area [83]. Another report indicates that FUS BBB opening facilitated brain accumulation of systemic etoposide with improved therapeutic effect [84]. While improved brain accumulation of small molecules is significant and encouraging, the delivery of larger biological drugs such as monoclonal antibodies is more challenging. In a recent study by Sheybani et al. CD47 blocking antibody was radio-labeled with 89Zr and injected systemically either before or immediately following FUS BBBO in mice. Accumulation of the antibody in the brain improved modestly when injected after FUS but did not change when it was injected prior to FUS [85].

While brain cancer is the most frequent application for FUS BBBO it has been used to enhance drug delivery in other models of neurological diseases. Kinoshita et al. used FUS to target delivery of polyclonal antibodies against the extracellular domain of the dopamine D4 receptor to the brain. Immunohistochemistry confirmed a positive signal of anti-rabbit IgG in the sonicated area in the ipsilateral hemisphere [82]. Alzheimer’s disease is another application where opening of the blood brain barrier can be exploited for either drug delivery or to enhance clearance of beta amyloid [86, 96]. A comprehensive review of applications for FUS BBBO in neurodegenerative diseases has been recently published [97]. An important tool available for achieving high precision of FUS BBBO is implementation of MRI-guidance (MRgFUS). FUS systems integrated with pre-clinical MRI are available and allow for excellent control and planning of the brain territory targeted for BBBO with subsequent immediate verification of its effect with contrast-enhanced T1 MRI. Indeed, MRgFUS has been widely used to enhance anti-cancer drug delivery to pediatric brain tumors in mice [87] or polymeric nanoparticles in healthy rats [35, 88]. One concern with FUS BBB opening is the need to titrate the energy of FUS and the dose of microbubbles to achieve optimal BBB opening but without causing damage. Indeed, complications of FUS BBBO have been reported including microhemorrhages and neuroinflammation [35]. There are several safe and effective blood brain barriers opening techniques and the choice of the optimal technique will depend on the specific application. Endovascular techniques, such as intra-arterial mannitol-based BBB opening, are ideal when relatively large brain volumes are targeted. They offer the unique advantage of local intra-arterial administration of the drug as the catheter is already in place. Another important feature of osmotic techniques is that the BBB opening half time is rather short in the range of 10–15 min. FUS has the main advantage of outstanding spatial control over the BBB opening area. However, targeting larger volumes of the brain is time-consuming and complex as the dosing of microbubbles has to be adjusted continuously to avoid damage to the endothelium. BBB opening half-time for FUS-based technique varies in published reports from several hours to days but is certainly longer than osmotic techniques thus may be suitable for systemic drug administration to smaller targets in the brain.

## Clinical implementation and theranostic strategies for BBB modulation

**Fig. 2:**
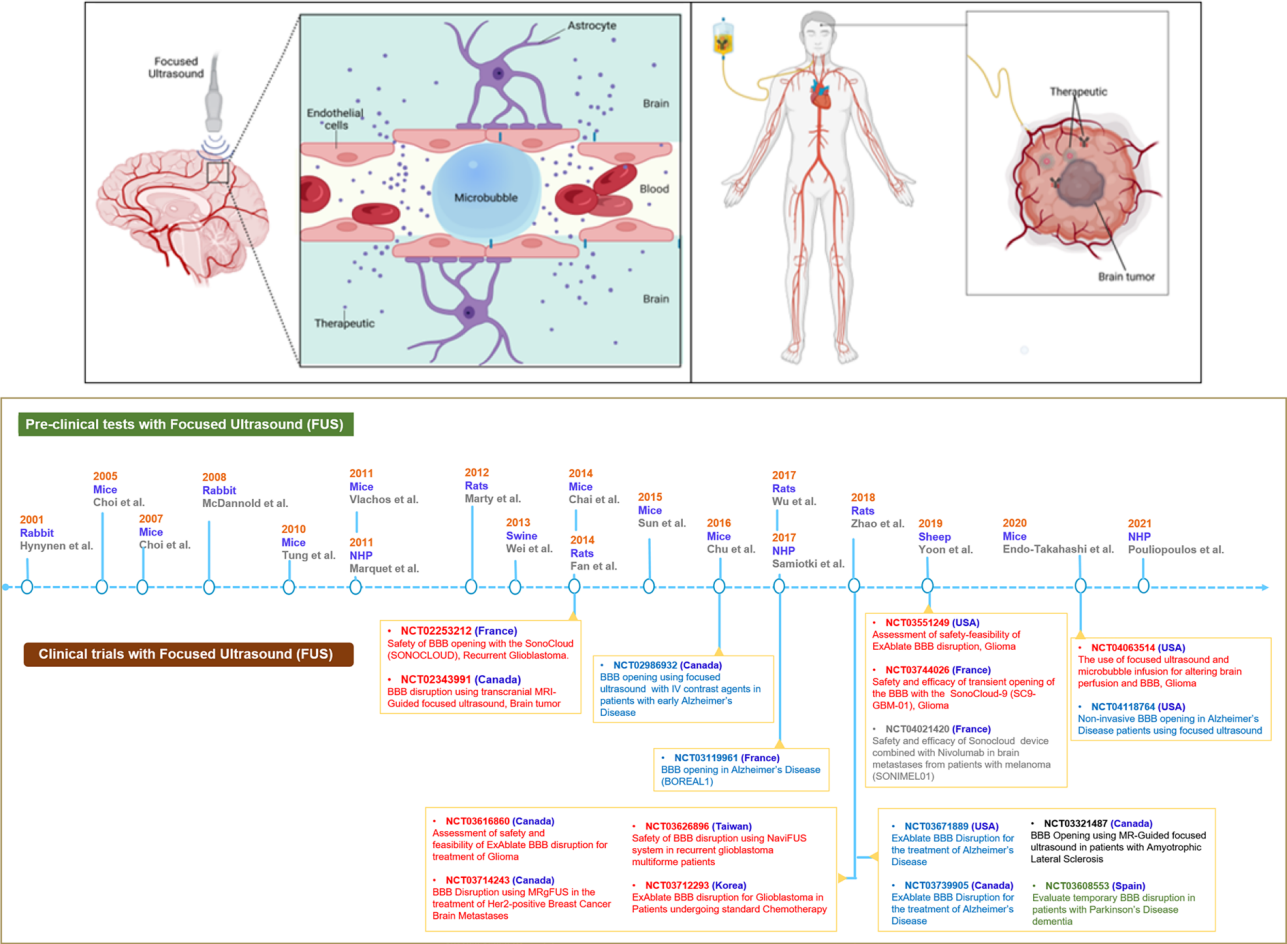
Clinical implementation and theranostic strategies for BBB modulation. Intra-arterial administration (upper section), preclinical and clinical trials using Focused Ultrasound (lower section) are illustrated

Multiple methods for BBB opening have been explored, however, all of them showed limitations in terms of their successful implementation in the clinical context [98–100]. Among these methods, sufficient information regarding the mechanism of action and safety aspects have been gained for the use of FUS and intra-arterial infusion. In particular, FUS has emerged as a promising non-invasive approach with proven success in preclinical models and encouraging results in clinical scenarios (Fig. 2**,** adapted from [101, 102]). FUS harnesses the thermal and mechanical effects of ultrasound focused on a specific area by a lens or transducer with the aim to minimize off-target effects. Application of FUS causes oscillation of bubbles in response to ultrasound waves, facilitating the mechanical opening of the BBB without injury to the vessels or brain tissue. A tentative two-decade long standardization process involving small (mice, rats) and larger preclinical model organisms (rabbits, sheep, swine, non-human primates) has led to the translation of FUS into the clinical use, including neurological (glioblastoma, Alzheimer's disease, Parkinson's disease) and other pathologies (metastatic melanoma, amyloid leukemia). The suitability of FUS technology to transiently increase BBB permeability and to increase passing of anticancer drugs [103], antibodies [104], neural stem cells [105], AAV-based vectors [106], nanoparticles [107], and chemotherapeutics [108] has been confirmed. In preclinical glioblastoma (GBM) models, FUS slowed tumor growth and improved survival rates [109–113]. Furthermore, chemotherapeutic agents such as carmustine, doxorubicin, and carboplatin have been tested in animal models of gliomas with FUS disruption of the BBB [109]. FUS has been demonstrated as a reliable approach to improve local chemotherapy and antitumor immune response in gliomas [114]. As a methodological advancement, both safety and feasibility of MRgFUS with intravenously injected microbubbles have been considered in patients with gliomas [115] and Alzheimer's disease [116]. A recent single-center study using repeated MRgFUS treatment for malignant brain tumors with a standard chemotherapy protocol showed no significant adverse effects (clinicaltrials.gov, NCT03712293) [117]. Similarly encouraging results were obtained in a recent clinical trial involving six patients with early Alzheimer's disease (AD) who tolerated a total of 17 FUS treatments with no adverse events and no cognitive or neurological deterioration [118]. Though FUS holds the potential to play a central role in non-invasive delivery of therapeutics, most clinical data come from trials with small patient numbers, which lack a prolonged follow-up period. The results of ongoing clinical trials will be critical for determining the suitability of FUS in humans. Compared to FUS, intra-arterial infusion for drug delivery is a relatively old approach, first described in the 1950s for the treatment of brain tumors [119]. Intra-arterial infusion of therapeutics increases the concentration of drugs delivered to the brain whilst minimizing systemic side effects. For this technique, a small catheter is inserted into the femoral artery in the leg, threaded through the body and into the brain where the drug is released.

Interestingly, intra-arterial administration is still considered far superior compared to other contemporary methods (e.g., oral or intranasal administration, intravenous or intracerebral injection), especially from a physiological perspective. Considering the preferential retention of administered substances in brain tissue, pharmacokinetic optimizations have favored intra-arterial over intravenous delivery approaches [120, 121]. A recent phase 1 study of intra-arterial administration of bevacizumab and cetuximab with BBB interruption in 13 children with high-grade glioma and diffuse intrinsic pontine glioma showed encouraging results [122]. Similarly, a phase I trial of intra-arterial administration of autologous bone marrow-derived mesenchymal stem cells in patients with multiple system atrophy appeared to be a safe and promising neuroprotective strategy [123]. Likewise, the results of a phase I/II clinical trial using repeated administration of intra-arterial bevacizumab after BBB disruption in newly diagnosed glioblastoma patients showed better progression-free survival and overall survival [124]. An interesting study addressing technological advances to improve delivery of AAV vectors to the brain suggests that intra-arterial delivery routes specifically with mannitol may provide significant advantage [125]. Rechberger et al. analyzed preclinical and clinical research findings on intra-arterial drug therapy for brain tumors and found that most studies were clinical in nature, with chemotherapy being the most common therapeutic modality and transient BBB disruption using mannitol was the most frequently investigated [126]. Based on this knowledge, clinicians are currently engaged in optimizing strategies to improve intra-arterial treatment for brain tumors and patient survival [127, 128]. This is further evidenced by the fact that intra-arterial delivery has been combined with imaging modalities (X-ray, CT scan, PET, SPECT, MRI, DC-EEG, etc.) to guide drug perfusion and to predict therapeutic effects [127, 129]. Despite this long history of intra-arterial infusion, its limitations, such as the risk of microembolisms, reactive immune responses, neurotoxicity and vascular toxicity remain a challenge. Furthermore, brain tumors are heterogeneous and highly vascularized near the periphery, limiting the delivery of drugs to certain regions of the tumor. Surprisingly, despite sufficient knowledge of intra-arterial infusion with FUS, their combination to improve the drug delivery across BBB has not yet been explored.

## Drug formulations to increase their BBB permeability

### Small molecule modifications

Substantial efforts have been made to tune properties of therapeutic agents to facilitate their penetration across the BBB. Approaches used to breach the BBB strongly depend on the size of the molecule to be delivered. Some small molecules are capable of passively diffusing through the BBB, and there is intensive work to improve their properties in this regard. Other small molecules use active systems in order to pass through the BBB. Large molecules are not able to passively cross the BBB, thus approaches have been made to fit large molecules into existing transport systems. Cellular therapeutics require diapedesis to extravasate to the brain parenchyma, and there are several ways to achieve this: through genetic, epigenetic, chemical and physical engineering and pre-conditioning of potential cellular products. Noteworthy, the penetrating therapeutic agents also need to avoid being actively effluxed back to the circulation by a guarding system of pumps and transporters. Thus, the biological, chemical and physical barriers are complex and delivery of a wide range of therapeutic agents to the brain remains difficult. Efforts towards increasing penetration of small molecules are centered around three mechanisms: increased diffusion, decreased efflux and better exploitation of transporters [99]. The lipophilicity is a critical property of small molecules, which make them amenable to passive transport through diffusion [130]. Additionally, molecular weight up to 400–600 Da and up to 8 hydrogen bonds in a molecule are characteristics that allow passive diffusion [131]. Methods exist for calculating the BBB permeability of small molecules [132] and artificial intelligence has been used to identify molecules able to cross the BBB [133]. The real-time feedback on small molecule biodistribution is an attractive but challenging strategy to better understand the dynamics of drug penetration and clearance from the brain. Small molecule-based fluorophore-drug conjugates have been developed, which are currently used in small animal studies [134]. Radiolabeling of small molecules allows visualizing drug dynamics in large animals and patients. However, radiolabeling is quite cumbersome as it requires radiosynthesis and typically access to the cyclotron to detect the 11C radioisotope, thus its widespread application is limited [135]. Some small molecules could be fluorinated thus presenting an opportunity for radioisotope with longer half-life. Detection of 18F isotopes can be achieved using commercial sources, which circumvents the need for an on-site cyclotron [136]. Overall, despite new directions, old challenges persist in small molecule delivery to the brain [137]. The small size of molecules also frequently limits their therapeutic potency, which prevents achieving a cure. It is compelling to continue research on small molecules penetration to the brain as they have an encouraging cost and access profile [138], however, we need to take into consideration the limitations of these small molecules. These include the types of drugs available and their therapeutic efficacy and specificity, thus small molecules will not be a standalone therapeutic solution for brain diseases. We will discuss other types of therapeutics to be considered in the following sections.

### Macromolecule modifications

While drugs with a large molecular size are unable to cross the BBB, several potential strategies exist to facilitate their delivery into the brain. These include: (A) pharmacologic formulation, such as exosomal encapsulation or cellular carriers for transcytotic transport across BBB; (B) conjugation with ligands for biological transporters and receptors in the BBB and (C) temporary disruption of BBB as discussed earlier. Transcytosis is a process of transport of large proteins, exosomes, microbes, viruses, bacteria or mammalian cells such as immune cells into the brain [139]. The efficiency of synthetic and biological drug carriers depends on their physico-chemical characteristics, such as particle size, surface charge, hydrophobicity, shape and elasticity. In general, properties limiting kidney clearance and extending time spent in the systemic circulation benefit the uptake of drug carriers by various organs including the brain. However, likely due to the relatively thick vascular membrane, brain penetration favors particles with a diameter smaller than 100 nm and a rod-like rather than spherical shape [140, 141]. Synthetic formulations for drug delivery to the brain have been extensively reviewed elsewhere and include liposomes as well as lipid-nanoparticles often stabilized using polyethylene glycol (PEG) or proteins such as albumin [142]. Exosomes are extracellular vesicles (40–160 nm in diameter) that are commonly produced by many cells and carry various nucleic acid, protein and lipid components of the cells of origin [143]. Exosomes gained attention as a potential vehicle for drug delivery to the brain after a breakthrough study demonstrated exosome-dependent and targeted delivery of therapeutic siRNA into neurons, microglia and oligodendrocytes using intravenous administration [144]. Despite significant progress in the characterization of exosomes the application of exosomes in therapy of CNS diseases still faces considerable challenges. This is due to difficulties in manufacturing at larger scale and standardization of exosomes, low yield, complexity of drug loading and difficulty in targeting exosomes to cells of interest [145]. These challenges could be mitigated, at least partly, by the use of well-established cellular drug delivery systems such as mesenchymal or neural stem cells that are known to secrete large amounts of exosomes [146, 147]. Neural stem cells (NSCs) seem especially suited for the application in cancer therapy due to their tropism to hypoxic tumor areas. NSCs are capable of delivering chemotherapeutic prodrugs, oncolytic viruses and therapeutic antibodies into brain tumors [147]. A recent study demonstrated that NSCs loaded with immunotherapeutic antisense oligonucleotides (ASO) accumulated in intracranial gliomas and delivered exosome-encapsulated cargo to tumor-associated immune cells [148]. Despite reports of certain ASOs crossing BBB using an unknown transporter, the majority of oligonucleotides undergo rapid kidney clearance and do not accumulate in the brain or brain tumors [149, 150]. The conjugation of oligonucleotides or carrier particles with ligands for BBB receptors or transporters has been widely explored. The transferrin receptor gained attention as it is expressed by brain endothelial cells. It was targeted using a variety of ligands including transferrin, ferritin, monoclonal antibodies and aptamers [151]. Low density lipoprotein (LDL) receptors have been targeted using lipid or silica nanoparticles modified with apolipoprotein E (APoE) or Angiopep-2 [152, 153]. Peptides such as rabies virus glycoprotein (RGD), TGN peptide and vascular cell adhesion molecule 1 (VCAM1) binding peptide were successfully used to deliver antibodies, nanoparticles, liposomes and exosomes into the brain in pre-clinical models and translated into several clinical studies [139]. Although none of these strategies has yet received FDA/EMA approval, the broad spectrum of technologies being tested and the intensive interest of both, academic institutions and pharmaceutical companies, underscore the chances of clinical translation of BBB targeted drugs in the near future.

### Cell modification to enhance their BBB crossing after grafting

Stem cell therapies for neurological diseases are challenging to deliver to the brain due to the barrier functions of the BBB. The poor transport of exogenous cells across the BBB limits the efficacy of intravascular administration. How stem cells migrate across the BBB is a controversial topic. Still, there are many reported similarities to immune cell infiltration, including rolling on and adhesion to the endothelium and transmigration across the BBB. The BBB becomes compromised during brain inflammation and injury, and cellular trafficking through the BBB is significantly upregulated [154]. Circulating leukocyte extravasation through the BBB is characterized by a multistep adhesion/migration cascade [155]. We developed in vitro microfluidic assays to analyze the interactions of flowing stem cells with a surface of endothelial cell-coated microfluidic channels. We noticed that human glial restricted progenitors (GRPs) or bone marrow mesenchymal stem cells (BM-MSCs) infused into microfluidic channels were simultaneously tracked, and the entire flow and docking phases were captured, including rolling, arrest, and crawling [156–158]. However, despite the observed process paralleling the mechanism used by leukocytes, the number of stem cells that docked to the vessel wall in in vitro microfluidic channels was limited. In this context, increasing diapedesis of transplanted cells is indispensable for cell transmigration in vivo and an important topic to study.

**Fig. 3:**
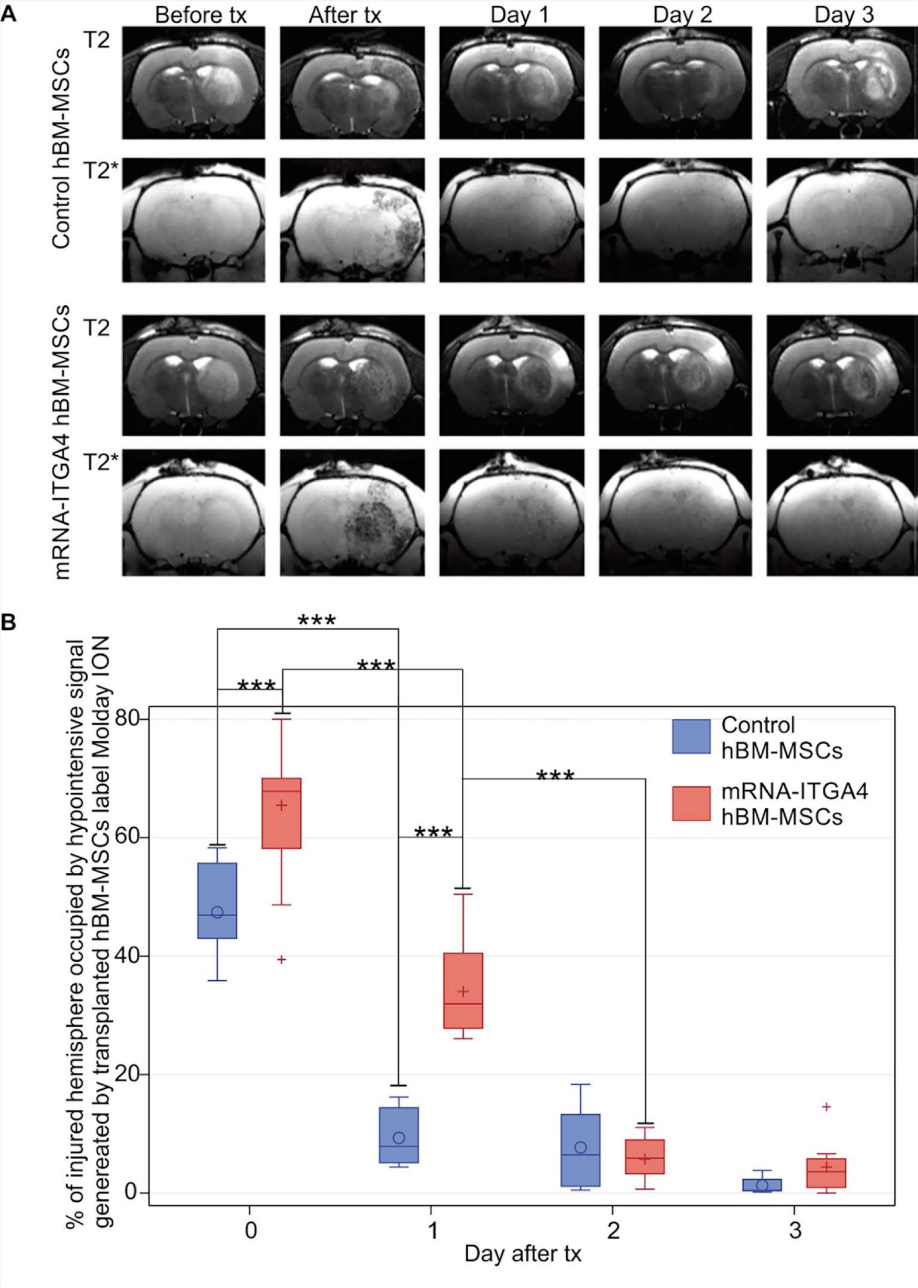
Evaluation of the presence of IA transplanted mRNA-ITGA4 transfected or control (naïve) hBM-MSCs in the rat brain subjected to focal brain damage using MRI scan assessment. **A** mRNA-ITGA4 transfected and control hBM-MSCs labelled with Molday ION were visible in MRI in T2 and T2* scans up to three days after transplantation (tx). **B** The box-plot graph shows the percentage of right hemisphere occupied by hypo-intensive signal generated by transplanted mRNA-ITGA4 transfected (red boxes) or Control hBM-MSCs (violet boxes). The type III fixed effects test was used to determine statistical significance, and the LMS method was applied to compare between groups and time points. Box charts present the dispersion and the shape of the data distribution for the test value in the compared populations. The length of the bars is equal to the quarter range (Q1–Q3) of the data, the tips of the mustaches indicate the minimum and maximum values, the line inside of the bar determines the median, while the circle/plus the arithmetic mean, the outliers are presented in the form of circles/pluses; *p < 0.05, **p < 0.01, ***p < 0.001 (n = 6). Reprinted from [162]

The adhesion molecule-dependent process of diapedesis described in leukocytes has been long recognized. Leukocytes extravasate through the ligand-receptor interactions. Among them, the very late antigen (VLA)-4–VCAM1 axis is a well-known contributor to the diapedesis of leukocytes. VLA-4 is expressed on the surface of cells, while VCAM1 is present on the endothelium. The VLA-4/VCAM-1 axis and its role in the diapedesis of transplanted stem cells have been described by Gavina et al. [90]. They demonstrated that migration of intra-arterially infused human CD133b stem cells into the muscles of dystrophic mice was dramatically reduced by the VCAM-1 blocking antibody. Similarly, the involvement of the VLA-4-VCAM-1 axis in the homing of stem cells was also reported by Brunner et al. [159]. In line with this, blocking VCAM-1 molecules by neutralizing antibodies significantly reduced bone marrow stem cell migration to the diseased heart in virus-induced dilated cardiomyopathy (DCM). Likewise, Jin group has shown the role of VLA-4 molecules in cell transmigration from the vascular bed to the tissue. In their studies, the intra-venous injection of VLA-4-expressing bone marrow progenitor cells CD34þ cells in tumor-bearing mice were effectively homed to the tumor and the antagonist of integrin a4/b1 reduced this homing [160]. The expression of VLA-4 receptors as docking molecules on the membrane of intravascularly transplanted cells seems to be also crucial for crossing BBB. Indeed, it was shown that NSCs sorted for the high expression of VLA-4 adhesion molecules more effectively migrated to the area of stroke in mice after intra-arterial delivery [161]. A significantly higher number of NSCs were found in the ischemic hemisphere of animals receiving NSCs-VLA-4(+) compared with NSCs-VLA-4(−). In further studies, overexpression of VLA-4 in human GRPs, obtained through DNA plasmid-based genetic engineering, increased the binding of transfected cells to cerebral endothelium after their infusion into a carotid artery in a rat model of global inflammation, compared to naive GRPs [156]. Jablonska et al. confirmed these observations. The authors demonstrated the efficient adhesion of transplanted, primaryVLA-4þGRPs transplanted i.a. (intra-arterial) to the cerebral endothelium of ipsilateral hemisphere in a middle cerebral artery occlusion (MCAO) rat model of stroke. The number of docked GRPs with high VLA-4þ expression was three-fold higher compared to naive GRPs with unmodified VLA-4þ expression. Moreover, the studies identified some infused VLA-4þ-GRPs extravasating through the blood vessel wall into the brain parenchyma whereas all naive GRPs remained inside the blood vessels [157]. Recently, it was shown that human BM-MSC transfection with VLA-4 molecules improved cell docking to blood vessels in the brain after infusion of BM-MSCs into the carotid artery of rats with focal brain ischemia [162]. Overexpression of VLA-4 in human BM-MSCs has been established by mRNA-based cell engineering [163]. The presence of VLA-4 proteins in BM-MNCs was transient and lasted for up to 24 h after transfection. Membraneous location of VLA-4 receptors on transfected MSCs improved the initial cell settlement to cerebral vessels in the injury area and increased their uptake into the brain visible in MRI scans (Fig. 3). However, MSCs with high VLA-4 expression remained inside the vascular lumen over the first two days. On the third day, nearly half of the MSCs present at the time extravasated from the cerebral vasculature to the perivascular space. Increasing VLA-4 expression on the cell surface to improve diapedesis after intra-arterial transplantation is a promising strategy. However, the number of cells that migrate from the cerebral vasculature to the brain parenchyma remain small and further studies on the recruitment of a higher number of transplanted cells are needed.

Another strategy to increase the transmigration of cells into brain tissue after intravascular infusion is to modify stem cells with factors that enhance chemokine receptor expression. Such a procedure has been shown to increase the number of cells homing to the brain. The critical role of the C–C chemokine ligand 2 (CCL2) and C–C chemokine receptor 2 (CCR2) in the targeted homing of stem cells was demonstrated by Guzman group [164]. After intracarotid delivery of NSCs in an experimental model of brain hypoxia/ischemia in mice, the authors observed significantly higher numbers of CCR2+/+ transfected NSCs recruited to the ischemic brain areas as compared to CCR2−/− cells, proving the importance of CCR2 for active homing of NSCs across the BBB. Modulating the expression of CCR2 in transplanted cells may offer a new way to improve the efficiency of intra-arterial stem cell therapy in the future. However, further investigation is needed to facilitate therapy with intravascularly infused exogenous stem cells.

## A few key considerations about the dynamics of the BBB

As aforementioned, the major concern in field is whether it is possible to open the BBB in a meaningful way without causing negative consequences. To achieve this, it is particularly important to broaden our understanding about the dynamics of the BBB. In this context, a few key considerations we proposed are: (1) whether the ensuing inflammation caused by BBB opening will be beneficial or detrimental to the brain microenvironment, (2) as the core structure of tight junction barriers are not static rather highly dynamic that allows discrete trafficking under physiological or pathological stresses, so whether areas of the BBB with different densities of tight junctions (lipid-protein composition) should be targeted as high-permeability gateways, (3) how can we empirically analyze the BBB based animal models to predict the human response accurately, (4) can we quantitatively model the interaction between BBB transport and glymphatic clearance (net fluid flow inward through arteries and outward through veins), (5) Whether transient transcriptional changes with long-term effects are to be expected, especially when conducting locus-specific BBB studies, and (6) since concentrations of several molecules in the CNS are subject to circadian oscillations/rhythms, therefore, we do need to check the permeability/efflux of our current compounds according to this circadian clock.

## Concluding remarks

Research into drug development targeting the CNS is complex and it is uncertain which approach will be successful. The systemic problems in the drug development industry are aggravated by the inaccessibility and sensitivity of brain tissue, while it remains the most suitable tissue to develop novel and innovative drug delivery systems. Despite the urgency of the field in addressing the problems in drug delivery to the CNS, methodologic divergences have been faced by the field for decades. The widespread use of animal models, the most commonly used model system in pre-clinical trials, is currently being questioned due to discrepancy in research findings from animals and human patients, particularly in BBB transposing systems. These translational problems have contributed to improvement of existing animal models and have fueled the development of human neurovascular models that mimic native neurovascularity more closely. In the search for in vivo models, tissue engineering has focused on tissue organoids, leading to the emergence of a burst of brain organoid models in recent years. Nonetheless, technological innovation and disease-modeling are currently still performed in assembled structures, using a bottom-up approach that has been used in the field for more than 30 years. In the next few years, hybrid strategies integrating disease-specific assembled structures and organoids-on-chip will become an integral part of pre-clinical and clinical research. This trend will guide the field towards effective precision medicine, with patient-derived organoids resembling the native tissue, integrated in a robust and reproducible BBB vascular network, achieving high statistical significance.

## Data Availability

Not applicable.
